# Digital and nurse-led support intervention in primary care during the first year after curative intent treatment for breast or prostate cancer: study protocol of two cluster randomised controlled pilot trials

**DOI:** 10.1136/bmjopen-2024-090848

**Published:** 2025-02-22

**Authors:** Ann Langius-Eklöf, Åsa G Craftman, Linda Gellerstedt, Nazmije Kelmendi, Kristina Rooth, Tina Gustavell, Kay Sundberg

**Affiliations:** 1 Department of Neurobiology, Care Sciences and Society, Karolinska Institutet, Stockholm, Sweden; 2 Academic Primary Health Care Center, Stockholm, Sweden; 3 Department of Upper Abdominal Diseases, Theme Cancer, Karolinska University Hospital, Stockholm, Sweden

**Keywords:** Breast tumours, Prostatic Neoplasms, Nursing Care, Self Care

## Abstract

**Introduction:**

The period directly after primary treatment for breast or prostate cancer is a time when patients feel unprepared about how to manage life and address unexpected health challenges. Supportive care should focus on identifying symptoms and concerns and involving survivors in their self‐care. Interventions using a blended model encompassing remote and in-person components may inform how supportive care can be organised. This protocol describes two pilot randomised controlled trials with the aim to investigate the acceptability, feasibility and potential effects of a 6 month digital and nurse-led support intervention in primary care for patients with breast or prostate cancer during the first year after primary treatment.

**Methods and analysis:**

Two cluster randomised pilot trials including patients with breast or prostate cancer during the first year after ending primary treatment will run from 2023 in primary care centres in Region Stockholm. The trials will have an estimated sample size of 20 patients in each arm. The intervention groups receive a digital and nurse-led support intervention in combination with standard care, and the control groups receive standard care alone. To assess acceptability and feasibility, the participants in the intervention groups and the study nurses will be interviewed. Furthermore, digitally logged data and field notes by study-specific nurses will be analysed. Data collection for the potential effects of the intervention is conducted through self-reported standardised and validated questionnaires at baseline, and at 3, 6, 12, 18 and 24 months. Data entry and analyses will be blinded to the researchers. Qualitative data will be analysed with content analysis, quantitative data will be evaluated by comparing changes within and between groups.

**Ethics and dissemination:**

This project was reviewed and approved by the Swedish Ethical Review Authority. Study results will be published in peer-reviewed journals and presented at scientific and professional meetings.

**Trial registration numbers:**

ClinicalTrials.gov, NCT06471452 and NCT05100121.

**Strengths and limitations of this study**

- The two pilot studies are cluster randomised and have a prospective, repeated measure design.
- The rigorous design is underpinned by the Medical Research Council framework for complex interventions and the use of patient interviews, clinical guidelines and literature reviews.
- The digital nurse-led intervention includes the use of an interactive app previously tested in randomised controlled trials with positive results.
- The acceptability and feasibility measures in the pilot trial will offer insight into whether a future definitive trial can be done and into the recruitment process.
- The pilot study sample may be too limited in terms of size and geographical sites to be representative and to capture a potential effect of the intervention.

## Introduction

### Background

 Breast and prostate cancer account for more than half of all cancer incidences and are the most common form of cancer in women and men in Sweden.^1^ Advances in the treatment of breast or prostate cancer have improved rates of survival and quality of life, but many patients have long-lasting symptoms and concerns in the aftermath of treatment.^2^,^5^ Primary treatment for breast cancer is commonly surgical excision followed by radiotherapy and/or chemotherapy depending on the type of tumour.^6^ In addition, endocrine therapy is frequently prescribed over a long period, up to 10 years, often rendering long-term side effects such as lack of energy, hot flashes, sleep difficulties and joint pains.^7^ Difficulties in handling side effects can lead to non-adherence to prescribed treatment and thereby render a greater risk of cancer recurrence.^8^ After primary treatment for prostate cancer—surgical excision or radiotherapy—most men need regular surveillance, and some are prescribed long-term adjuvant antiandrogens. All treatments for prostate cancer may have a long-term impact on the patients’ quality of life and sexual, urinary and bowel functions.^9 10^

Many patients in the first year after primary treatment for breast or prostate cancer have unmet informational, physical, psychological and emotional healthcare needs.^11 12^ The period directly after primary treatment has been recognised as a time when patients feel unprepared about how to manage life and must address unexpected challenges, not knowing what to do about it, or where to seek assistance.^13^ Lingering symptoms and concerns remain after treatment for prostate cancer, and timely information and support are considered important by patients.^14^ Patients with breast cancer describe how they try to manage by themselves, but express a wish for extended and more individually tailored support.^15^

Supportive care should focus on identifying symptoms and concerns and involving patients in their self‐care to improve long‐term health.^16^ In Europe, there is no formalised indication of how supportive care should be organised, and many survivors after ending primary treatment are expected to be seen in the primary care setting.^16^ In Sweden, the basic health and medical care for most common conditions and illnesses is generally referred to as primary care. There are guidelines from the European Society for Medical Oncology (ESMO) showing that patient-reported outcomes should be used in the clinic to include any aspect of a patient’s health status that comes directly from a patient.^17^ Symptom self-monitoring in the continuum of cancer care using electronic patient-reported outcomes (ePRO) is recommended by ESMO and should include questions for evidence-based symptoms, with the advantage of automated alerts to clinicians for worsening symptoms. Studies integrating ePRO to support patients in clinical practice with cancer during treatment have shown promising results such as decreased symptom burden and increased health-related quality of life (HRQoL)^18^,^20^ and survival^21^ in comparison to control groups. Studies also show high user engagement as well as feelings of security and satisfaction with easy access to caregivers.^22 23^ Research findings in a review article disclose a considerable variation in the features offered in the different symptom reporting systems.^20^ The review shows that over half of the systems had the facility for healthcare providers to monitor patient data over time, while fewer than half included the facility for patients to monitor and review their own data. Furthermore, less than half had general patient information about cancer treatment and side effects, and only one-third had automated patient advice on symptom management. Another recent review of digital health studies showed that positive outcomes were shown when the intervention targeted symptom reporting and self-care functions in combination, but only a few studies had also provided personal interaction.^24^ Optimal supportive care goes beyond just using ePRO. While ePRO plays an important role in the assessment of outcomes, it is essential that these outcomes are appropriately managed in clinical practice.^25^

Nurse-led interventions in cancer care, reported in an overview of reviews, comprised a combination of educational and psychosocial interventions.^26^ The review did not disclose if the studies were performed during or after treatment. The effectiveness of the interventions was inconsistent but significant when supporting patients’ coping, which included symptom education, problem‐solving therapy and coordinating and monitoring the treatment. Another review focusing on characteristics of care models for cancer survivorship describes that models using technology to enhance remote interaction between patients and healthcare providers improve the quality and effectiveness of care.^27^ Many of the care models used simple methods of communication such as websites, telephone calls and SMS messaging, and they position technology as a practical means to increase provider capacity and improve access to care. A minority of the described interventions used a blended model encompassing both remote and in-person components. The blended models were better suited for interventions detecting potential complications that may require clinical support. The duration of the interventions varied, and it was concluded in the review that, in studies of short duration, it was difficult to ascertain whether patients and providers had engaged with these models long enough to derive sustained outcomes. Studies reporting on nurse-led support interventions for patients with cancer, specifically in the context of primary care, are few. A pilot study to promote self-care for prostate cancer survivors showed that an educational nursing intervention was feasible and that the men gained some positive health outcomes.^28^ The intervention included an initial face-to-face appointment with follow-up visits as agreed. The nurse-led sessions were not based on ePRO, and there was an inconsistency in the follow-up visits, whereas less than one-third of the patients returned for a second or a third follow-up.

Supportive nursing interventions in cancer care have shown to be effective to varying degrees. The evidence is not conclusive, perhaps due to the complexity of nursing interventions with numerous components. The literature describes a variation of deliverance, and the shortcomings of such interventions and the number of studies conducted during the first year after primary treatment are unclear. Interventions involving ePRO are demonstrating rapid growth and have been shown to be helpful and effective for symptom management and the supportive care of patients with cancer. Intervention studies where ePRO and in-person support are simultaneously offered are still in their beginning stages. Also, in this field of research, reviews identify gaps in how to integrate ePRO for best clinical practice. The period directly after primary treatment is a time when patients feel unprepared and unsure where to seek care, whereas the primary care centres (PCCs) are often the given option. We seek to find out whether an ePRO intervention in combination with nurse-led support in the primary care setting is viable or in need of modifications ahead of a larger-scale evaluation of effectiveness. These parallel-running pilot RCTs are the first steps of preparation towards larger trials to deliver an intervention that includes important aspects as suggested in the literature.

### Objectives

The aim is to evaluate the acceptability, feasibility and potential effects of a digital and nurse-led support intervention in primary care for patients with breast or prostate cancer during the first year after primary treatment.

## Methods

The protocol is prepared according to the SPIRIT 2013 statement^29^ and adapted with items from the CONSORT 2010 statement^30^ based on a guide to the reporting of protocols of pilot and feasibility trials.^31^ The SPIRIT schedule of enrolment, intervention and assessments is employed (table 1).

**Table 1:** The SPIRIT schedule of enrolment, intervention and assessments

Timepoint	Enrolment	Allocation		Post-allocation			
	-*t*_1_	0	3 months	6 months	12 months	18 months	24 months
Enrolment:							
Eligibility screen	X						
Invitation letter	X						
Informed consent	X						
Allocation to intervention or control group		X					
Intervention: Reporting in Interaktor and health dialogues							
Breast cancer cohort		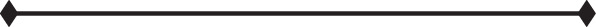					
Prostate cancer cohort		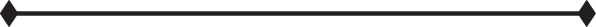					
Assessments:							
Demographic and medical data		X					
Primary outcomes				X			
Secondary outcomes		X	X	X	X	X	X

### Patient and public involvement

The intervention tested in these trials was developed in response to our previous research showing that patients treated for breast or prostate cancer have extended needs of support from the healthcare providers beyond the completion of treatment.^14 15^ The technique for ePRO and digital support is a system (Interaktor) including an app and a web interface. Interaktor was developed in cooperation with patients and healthcare professionals through interviews. For this follow-up project after cancer treatment, the app has been tested in a small sample of patients with feedback that has guided refinements before the final model.

### Study design

The study design is underpinned by the Medical Research Council (MRC) framework for complex interventions.^32^ These studies have cluster randomised controlled pilot trial design including patients with breast and prostate cancer, respectively, during the first year after ending primary treatment. The two pilot RCTs will be performed in PCCs in Region Stockholm. The studies have two parallel arms: the intervention plus standard care (intervention group) and standard care alone (control group). The intervention involves an entire PCC, and cluster randomisation is used to avoid the spill-over effect.^33^ To achieve representativeness for the cluster randomisation, the Care Need Index (CNI) for calculating economic compensation to the PCC is used.^34^ CNI measures healthcare needs for the distribution of primary care resources to populations with the biggest need. A high CNI equals low socioeconomic status, and a low CNI equals high socioeconomic status. Ten PCCs in the region were considered sufficient as a basis for our sampling. Five pairs of PCC were matched to the CNI and the size of the PCC regarding a number of allocated patients. Subsequently, a randomisation through sealed envelopes allocated the PCC to either intervention or control in each pair.

### Participants

#### Enrolment

The recruitment and participant enrolment were started in October 2023 and is expected to continue until January 2026. Patients diagnosed with breast or prostate cancer and who have ended primary treatment are identified through the database at the Stockholm Centre for Health Data on healthcare consumption by combining PCC individual identification codes and relevant ICD-10 codes. Criteria for inclusion are completed primary treatment with curative intent for breast or prostate cancer within the past 6 months, >18 years and able to speak and understand Swedish. The criterion for exclusion is cognitive impairment. The plan is that the intervention can be given during the first year after primary treatment and that the intervention period of 6 months falls within the frame of the first year. Eligible patients in the intervention PCC and the control PCC are consecutively recruited by an invitation letter prepared by the researchers and posted from the PCC. Patients who agree to participate return an informed consent form directly to the researchers (online supplemental material). A reminder is sent within 2 weeks to those who have not been heard from. The patients are then contacted by the researcher who estimates the possibility of inclusion with regard to the inclusion criteria.

### Standard care in the study region

After treatment for breast or prostate cancer, all patients have scheduled visits to the oncological clinics for regular follow-ups according to guidelines for respective diagnoses. For breast cancer, annual follow-ups are usual for at least 5 years and include mammography and in many cases endocrine therapy.^35^ For prostate cancer, follow-up is carried out at 3 or 6 months during the first year and includes a PSA test.^36^

All patients with breast or prostate cancer should be offered a coordination contact nurse at the oncological clinic to call when needed.^37^ The purpose is to improve information and communication between the patient and healthcare service and strengthen the patient participation in care. In Sweden, the basic health and medical care for most common conditions and illnesses is generally referred to as primary care.

Primary care has no specific role in caring for patients in the follow-up of cancer treatment. Patient contact with primary care occurs on the patients’ initiative when seeking care for any health concerns.

### Intervention

#### Intervention delivery

**Figure 1:**
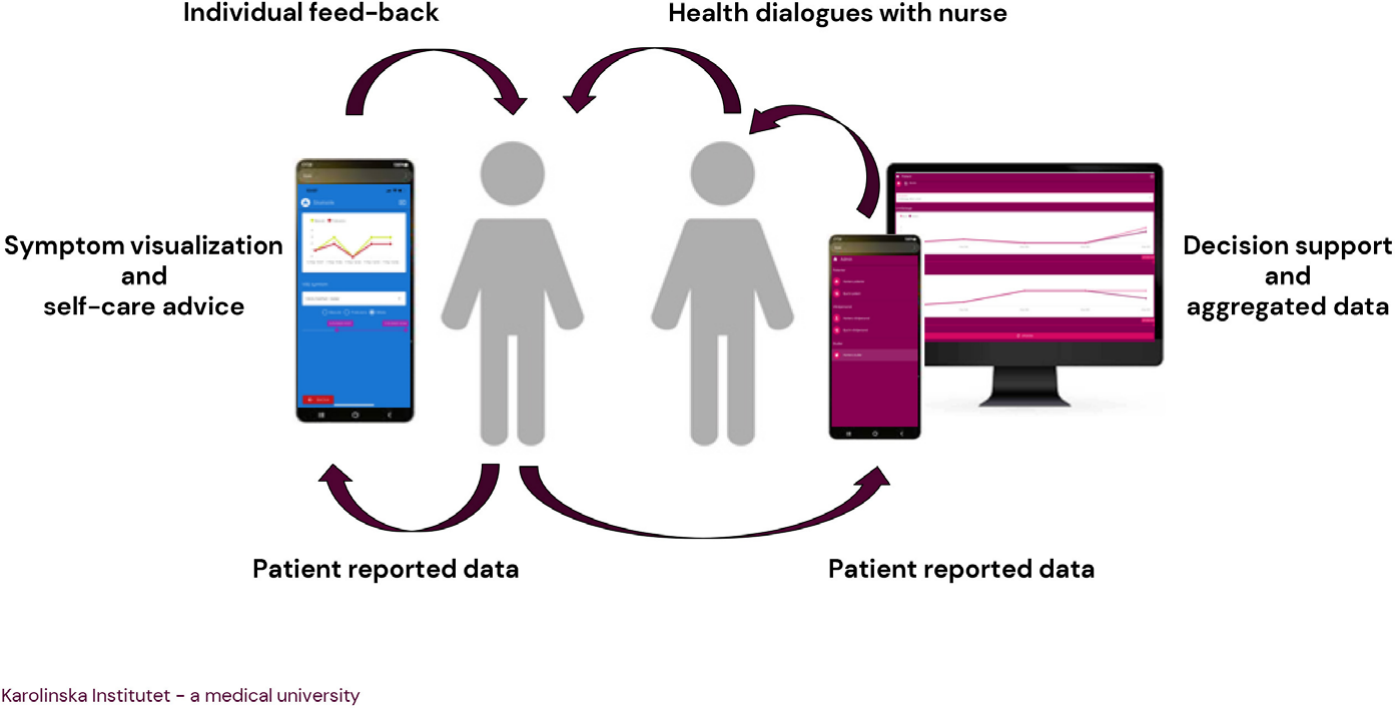
Illustration of the intervention.

The intervention is intended for a 6 month period and consists of patients using an app for symptom reporting and self-care advice, together with supportive health dialogues with a study nurse at the intervention PCC (figure 1). The nurse is employed at the PCC and experienced in working within the primary care setting. The nurse receives a preintervention audio-recorded tutorial regarding symptoms and rehabilitation measures to assess for each diagnosis. Patients are expected to report symptoms regularly on a weekly basis or as agreed with the nurse. The nurse logs on to the web interface to view the reports before the patient’s visits. A study folder at each PCC provides information and flowcharts of the study process, consent forms, checklists for health dialogues and handbooks for patients and nurses, as well as a knowledge base and self-care advice. The nurses will have continuous contact with the researchers and access to technical support.

#### Digital nurse-led support

Interaktor is a digital system for symptom management used in our previous studies during treatment for cancer.^38^,^40^ The system includes (a) an app that is downloaded to the patient’s phone or tablet where they report symptoms, view the reports in graphs and have access to a knowledge base and self-care strategies and (b) a web interface for the nurse to monitor the incoming reports, available in real-time. The content of the app is based on literature reviews, interviews with patients and healthcare professionals and national guidelines for each diagnosis. In this study, the patients in the breast cancer group report joint pain, discomfort in the breast, numbness/tingling in hands and feet, hot flushes, dry mucous membranes, fatigue, sleeping difficulties, depression, worry, concentration and memory, appetite and sexual health. The prostate cancer group reports on bladder function (difficulties urinating, leakage, urine urgency, blood in urine), bowel function (loose stool/leakage, constipation, flatulence, blood in stool), swelling/lymphoedema, pain, hot flushes, fatigue, sleeping difficulties, depression, worry, appetite and sexual health. If the patient answers yes to experiencing a symptom, there is a follow-up question about the frequency of the symptom—not often, sometimes, rather often or very often—followed by their rating of the distress of the symptom—not distressing, a little distressing, rather distressing or very distressing. The question concerning appetite is only assessed by the level of distress, and the question about sexual health is assessed on occurrence, allowing for commentary. Before sending in the report, the patients can write a free-text message. The app has an alert function where specific symptoms are set to generate an alert based on a risk assessment model. Alerts are triggered when patients answer that the symptoms occur *very often* and are *very distressing*. In the prostate cancer app, alerts can be triggered for pain, blood in stool, blood in urine and loss of appetite. In the breast cancer app, the reported symptoms are not considered acute and alerts are not generated. The triggered alerts send a notification to the nurse who is expected to contact the patient within 24 hours (during weekdays). The patients have continuous access to evidence-based self-care and lifestyle advice related to disease-specific symptoms. In connection with reporting the presence of a symptom, a notification shows the patient related self-care advice. These recommendations and general information about treatment and side effects (ie, what has caused them and how the symptom is experienced) can be retrieved in the knowledge base in the app. The recommendations for self-care and which symptoms should promote contact with a healthcare professional are the same as those provided through healthcare guidelines. Furthermore, reliable information can be retrieved through external links to relevant web pages or videos. Both the app and the web interface have a display of graphs to view the history of symptom reporting. Push notifications in the app will serve as reminders to report.

The reports made by the patients will be used in health dialogues, which are proposed to engage and support patients in self-care. Within a month after study inclusion, the nurse at the PCC books a meeting with the patient for a first dialogue. At this appointment, the nurse introduces the app and instructs the patients in the reporting procedure. The following health dialogue takes place in agreement between the patient and the nurse either as telephone calls, video conversations or face-to-face meetings. If needed, additional actions such as support from a physiotherapist, social worker, dietician or physician can be planned.

### Outcomes

#### Acceptability and feasibility

To assess acceptability and feasibility, the participants in the intervention group and the study nurses will be interviewed about their experience of using Interaktor and the health dialogues. Furthermore, field notes with reflections from the health dialogues will be taken by study nurses. Objective measures will be extracted from the logged data in the app, that is, the number of reports (adherence) and self-care advice viewings. After the intervention has ended (at 6 months), the patients are surveyed via a questionnaire including the Acceptability E-scale^41^ and two selected items from the System Usability Scale.^42^ The composed questionnaire assesses ease of use, understandability, enjoyability, helpfulness, time required and overall satisfaction. To evaluate the feasibility of a future trial methodology, enrolment, recruitment and withdrawal rates will be documented. Other feasibility measures include completion rates and missing data in each study questionnaire.

#### Potential effect outcomes

To evaluate the potential effects of the intervention and obtain estimates for a future full-scale trial, outcomes are collected through self-reported standardised and validated questionnaires at baseline, and at 3, 6, 12, 18 and 24 months after intervention has started. HRQoL and symptom distress are assessed by the European Organisation for the Research and Treatment of Cancer Quality of Life Questionnaire EORTC-QLC-C30^43^ including disease-specific modules QLQ-PR25,^44^ QLQ- BR45^45^ and EQ-5d-5L^46^; the Patient Activation Measure for knowledge, skills, beliefs and confidence of managing health and healthcare^47^; the Sense of Coherence Scale-13, which measures overall coping ability^48^ and communicative and critical health literacy (5-items).^49^ The quantitative data will be triangulated with interview data from patients to explore perceptions regarding the effects of the intervention.

Medical data will be obtained from patients’ medical records. Demographic data (age, education, marital/civil status, having children at home, employment status, sickness absence) and comorbidity are self-reported. For the data measurement plan, see table 1.

#### Data management and blinding

All data is securely stored in line with the university regulations. Logged data for primary outcomes is retrieved on the university server where the Interaktor system is hosted. The secondary outcomes are collected through self-reported questionnaires at baseline, and at 3, 6, 12, 18 and 24 months after the intervention has started. The participants receive postal questionnaires at all six time points and reminders if not returned after 2 weeks. Patients are not blinded regarding treatment allocation as they are randomised in accordance with allocation to a PCC. The researchers will be blinded at data entry and when analysing coded patient-reported data.^50^ To maintain blindness, a separate data sheet of the outcome measures will be generated without a record or group I.D.

### Sample size

We adopted a sample size calculation for pilot randomised trials using a CI approach.^51^ To evaluate the potential effect of the intervention as compared with the control, and assuming a medium standardised effect size (0.5), 12 participants are required in each group with 80% one-sided CI. The question ‘overall satisfaction with the system’ in the Acceptability E-scale was chosen as a primary measure of acceptability for the sample size calculation. Aiming for the intervention to be acceptable to 70% of the participants with 20% precision (at least 50% were satisfied to use the intervention), 18 participants are required in the intervention arm. With an estimation of a 10% dropout, the total sample size of 20 participants in each study arm for each respective study group was considered appropriate.

### Analyses methods

Descriptive statistics will be used to analyse logged data from the use of the app, and descriptive qualitative analyses will be used for interview data. For the comparison of the potential effect measures between the groups, inferential and comparative statistical analyses appropriate for data level and distribution will be used, and mixed methods analysis will be applied to integrate quantitative and qualitative data.^52^

### Monitoring

#### Data monitoring and auditing

On-site data management procedures comply with Good Clinical Practice. Data that are collected through postal questionnaires are stored according to university procedures. Logged data is stored in the university safe server and can be retrieved only by responsible researchers. The researchers take responsibility for following trial conduct in line with ethics, governance and policies. Data will be analysed after the intervention (when all patients have been included and ended the reporting and nurse-led dialogues) and any interim analyses are not planned.

#### Harms

The research described in this protocol is considered having ‘negligible or no risk’ for harm or discomfort for the participants. During the intervention, the nurses will take field notes for collecting, assessing, reporting and managing solicited and spontaneously reported adverse events and other unintended effects of trial interventions or trial conduct. Patients in both study arms will continuously receive standard care, which will typically mean clinical guidance if they contact the healthcare service for any reason.

## Ethics and dissemination

### Research ethics and approval

Ethical approval has been obtained from the Swedish Ethical Review Authority (record number 2019—00379_2023-02423-02). All patients to be included in the study will be given oral and written information underscoring the voluntary nature of participation. Those who agree to participate will sign a written consent form. Patients can at any time discontinue trial participation on their own request or in case of worsening disease.

### Data confidentiality

Logged data will be accessible only to researchers in the group, and nurses will manage reports and generate alerts when caring for the patient. Only the researchers in the group will be able to access data collected via questionnaires and interviews. The results will be presented in such a way that no participant can be identified. Interaktor is developed for research, and data are stored in a safe server at Karolinska Institutet. Data transmission and encryption are handled by the security protocol Secure Socket Layer.

### Ancillary and post-trial care

After the trial, both study groups will continue to receive standard care according to the national guidelines.

### Dissemination plan

Study results will be published in peer-reviewed journals and presented at scientific and professional meetings as well as in public popular scientific contexts.

## Discussion

This study protocol describes two parallel pilot RCTs for a digital and nurse-led support intervention in primary care for patients with breast or prostate cancer during the first year after primary treatment. Ongoing poor health and long-term rehabilitation are common concerns after treatment, and support should be prioritised to improve well-being, prevent recurrence and increase survival.^16^ Individuals who have finished treatment for cancer and experience health problems related to treatment are today often directed to primary care in between hospital follow-up visits. A recent interview study showed that cancer survivors experience that primary care services do not play a significant role for them.^53^ Lacking personal continuity and lacking commitment to cancer-related needs were presented as obstacles to their satisfaction. In a review by Mayo *et al*,^54^ cancer survivors express preferences for the organisation and delivery of supportive care in the post-treatment phase that fluctuates based on their perceived health needs. The authors suggest that the development of new survivorship healthcare models should consider survivors’ preferences and allow flexibility in care delivery to facilitate engagement, uptake and the effectiveness of support. Supportive care is much more than a series of incoherent services along with standard follow-ups; it is a conceptual framework guiding the planning, resourcing and delivery of cancer care.^55^ It needs to include early identification, timely intervention and multidisciplinary collaboration, reducing fragmentation and achieving outcomes that matter to patients.

Symptom monitoring to manage persistent or new symptoms can be useful in the post-treatment period of patients with cancer, but the evidence is so far limited.^17^ The outcomes assessed should be meaningful and clinically actionable, and there is a need to develop new ways of working together in the healthcare team to ensure an effective response to the data. We consider that an intervention such as Interaktor could benefit supportive care in the aftermath of cancer. In our previous studies with Interaktor during treatment for different cancer diagnoses, the result was shown to be very positive. The adherence was high for using the app daily during treatment, and the patients experienced the use of the app as easy and safe.^22 56^ At 2 weeks^38^ and 6 weeks^39^ after the end of treatment, patients rated better emotional well-being and less symptom distress than the patients in the control groups. Furthermore, using the app for self-care advice facilitated patient participation, and there was in some cases an improvement in health literacy and a higher perception of receiving individualised care.^57^

### Potential benefits and limitations

There are good reasons to believe that the routine collection of ePRO facilitates person-centred care where the patient is a participatory member of the team. Early identification of symptoms may guide symptom management and self-care ability, prevent more illness and lead to a faster return to daily activities and a better quality of life. Integrating this way of taking care of patients in need of support after cancer treatment in primary care could facilitate scheduling healthcare visits targeting the patients in need of surveillance.

This study protocol will inform the development of a future definitive larger trial. As a pilot RCT, in the protocol we have described primarily aims to answer questions of acceptability and feasibility. For this reason, we have not powered the study to formally test any hypothesis of efficacy or adequately understand sources of individual differences in intervention efficacy. The external validity is limited, although we propose that results from a larger trial may be transferable to other care settings for patients with other chronic diseases.

## supplementary material

10.1136/bmjopen-2024-090848 online supplemental file 1
