# Supplementary material for: Digital and nurse-led support intervention in primary care during the first year after curative intent treatment for breast or prostate cancer: study protocol of two cluster randomised controlled pilot trials
Source: BMJ Open. 2025 Feb 22;15(2):e090848. doi: 10.1136/bmjopen-2024-090848 (PMC11848687; doi:10.1136/bmjopen-2024-090848)
Supplement: online supplemental file 1 [file bmjopen-15-2-s001.doc]

|  |  |
| --- | --- |

**Information on Participation in a Research Study Regarding Patients Who Have Undergone Treatment for Breast Cancer/or Prostate Cancer (Intervention group)**

You are hereby invited to participate in a research study at Karolinska Institutet (KI) aimed at evaluating the effect of extended support at the primary healthcare center during the initial period after completing treatment for cancer.

The purpose of the study is to evaluate the effects of using a smartphone and tablet app for regular symptom reporting in combination with extended support from a nurse at the primary healthcare center regarding symptoms, quality of life, self-care ability. Comparisons will be made with a control group receiving standard care. In the study, healthcare centers will be randomized to have patients use the app in combination with supportive and standard care or to a control group receiving standard care.

Your primary healthcare center has been randomized to the group receiving the app and extended support (intervention group). If you agree to participate, this will mean that in addition to standard care, you will use the app for 6 months, where symptoms are reported to a nurse once a week up to once a month. Reporting symptoms takes a few minutes each time. The app also displays self-care advice on how to manage the symptoms you are experiencing. In addition to symptom reporting in the app, you will have conversations with the nurse regarding goals for your cancer rehabilitation based on what you have reported in the app.

The study also involves answering questionnaires regarding perceived quality of life, well-being, and self-care ability at the start of the study, and then at 3, 6, 12, 18, and 24 months thereafter. Medical data will be collected from patient records. At the end of the study period, you will be asked to be interviewed about your experiences with the care. The interview will be arranged in consultation with you and the lead researcher and is estimated to take about 30 minutes. It will be recorded to be transcribed anonymously for analysis.

Participation in the study is voluntary, and if you choose to participate, you can withdraw at any time without having to explain why and without it affecting your care within the healthcare system. Your current or future care will not be affected by whether you choose to participate or not

How Your Personal Data is Handled

For the conduct of the research study, KI will process your personal data (name, age, contact information, medical condition, and treatment). The data and the information you provide within the study are processed in accordance with the General Data Protection Regulation (GDPR) and are protected by the provisions of the Public Access to Information and Secrecy Act, which means that no unauthorized person will have access to the data. The responsible party for the processing of your personal data is Karolinska Institutet

You have the right to know what data about you is being processed within the study, where it was obtained from, and to whom it may have been disclosed. You can receive an extract showing this free of charge once a year upon written signed request addressed to the designated responsible researcher. If any information is incorrect, you have the right to have the information corrected, restricted, or deleted.

The results of the study will be published in a way that no individual participant can be identified. Your personal data and collected information will be archived for 10 years after the study has concluded and will then be destroyed. If you have any questions about how your personal data is handled, you can contact Karolinska Institutet’s Data Protection Officer (dataskyddsombud@ki.se). If you have any complaints about how your personal data has been handled, you can contact the Swedish Authority for Privacy Protection (imy.se).

By my signature, I give my consent to participate and for my personal data and other information about me to be processed as described. I agree that medical information related to the study may be obtained from my medical records and registers. I have been informed about the study and given the opportunity to ask questions.
