# Supplementary material for: Digital and nurse-led support intervention in primary care during the first year after curative intent treatment for breast or prostate cancer: study protocol of two cluster randomised controlled pilot trials
Source: BMJ Open. 2025 Feb 22;15(2):e090848. doi: 10.1136/bmjopen-2024-090848 (PMC11848687; doi:10.1136/bmjopen-2024-090848)
Supplement: Supplementary file 2 [file bmjopen-2024-090848.reviewer_comments.pdf]

PEER REVIEW HISTORY

BMJ Open publishes all reviews undertaken for accepted manuscripts. Reviewers are asked to complete a checklist review form and are provided with free text boxes to elaborate on their assessment. These free text comments are reproduced below.

ARTICLE DETAILS

Title (Provisional)

A digital and nurse-led support intervention in primary care the first year after curative intent treatment for breast or prostate cancer: study protocol of two cluster randomised controlled pilot trials

Authors

Langius-Eklöf, Ann; Craftman, Åsa G.; Gellerstedt, Linda; Kelmendi, Nazmije; Rooth, Kristina; Gustavell, Tina; Sundberg, Kay

---

VERSION 1 - REVIEW

Reviewer 1

Name Cai, Yingjie

Affiliation First Affiliated Hospital of Dalian Medical University, Department of Nursing

Date 04-Sep-2024

COI none

Thanks for your interesting work of supportive care for patients with cancer, I recommend you revise this paper as below:

1.In general, the logical expression of the article needs to be strengthened.

2.What is your research question? Research design should revolve around your research question.

3.Why did your research focus on breast and prostate cancer? Do the two groups have the same or similar nursing problems?

4. What is Primary treatment? The definition of primary care may vary from country to country. As an article published in an international journal, I suggest you to explain this in the article.

5.The study is designed as a randomized controlled trial. How to achieve randomization? It was not described in the registered clinical trial, it is recommended to describe it clearly in the article.

6.The description in this article differs from the registered clinical trials,

(1)The intervention period of this study is 6 months, but the duration of intervention for prostate cancer in the registered clinical trial was 12 months.

(2)Some of the secondary outcome measures in this paper were not involved in prostate cancer studies in registered clinical trials.

7.Page 7 line 3

'some studies with a smaller sample size and insufficiently sensitive assessment methods could not detect any changes in QoL. '

it is the limitation of current studies, about your study, have you solve these limitations?

Page 16, line 3, 'We have studied the use of Interaktor during treatment for different cancer diagnoses and the result was shown to be very positive...'

In this paragraph, apparently, your study also found no positive effects of the APP in terms of quality of life. Therefore, I suggest that you revise it to make the limitations of the existing studies relevant to your research question and study design.

8.Page7 line 53

'no studies combining ePROs and nursing support in primary care with a focus on improving patient symptom management and QoL'

The expression of this sentence is too arbitrary and it is suggested to be modified.

9.It is recommended to describe the admission criteria for research nurses.

**Reviewer**	**2**
**Name**	**Zhong, Jun**
**Affiliation**	**Zhongnan Hospital of Wuhan University**
**Date**	**12-Sep-2024**

COI **The reviewer declares that she has no known competing financial interests or personal relationships that could have appeared to influence the work reported in this paper.**

---

1. The objective mentions that the study population consists of those "in the first year after receiving primary treatment," but the actual intervention period is 6 months, and the follow-up lasts for 24 months. Isn't this inconsistent?

2. The inclusion criteria should be more specific. Will patients with comorbidities be included in the study?

3. Have you considered increasing the sample size?

4. The primary and secondary objectives are somewhat confused and unclear. The primary objective is to assess acceptability and feasibility, but the secondary objectives seem to overlap in scope, particularly regarding the evaluation of potential effectiveness.

5. The methods section lacks detailed descriptions of the randomization and blinding procedures conducted at the primary care centers.

6. The description of the digital support in the intervention is not detailed enough, particularly regarding the types of recommendations provided through the app and the criteria that trigger alerts.

VERSION 1 - AUTHOR RESPONSE

Comments to the Author:

Reviewer: 1
1.In general, the logical expression of the article needs to be strengthened.

Author response:

We have carefully reviewed the manuscript and made several amendments to improve its logical flow and structure. Additionally we have overlooked the language and phrases and made some revisions (not highlighted in red).

---

2. What is your research question? Research design should revolve around your research question.

Author response:

The research questions to be answered in these two trials is whether the intervention with Interaktor in combination with nurse- led support is acceptable and feasible in a primary care setting. For this we use cluster randomised controlled pilot trial design. The outcomes for acceptability and feasibility are described in the outcomes section (page 9). Further the pilot trial seeks to evaluate the potential effects of the intervention and obtain estimates and sample size calculation for a future full - scale trial.

We agree that this could be made more obvious and we have tried to clarify by rewriting the study rational (Introduction, page 5).

---

3. Why did your research focus on breast and prostate cancer? Do the two groups have the same or similar nursing problems?

Author response:

The choice to include patients with breast or prostate cancer is based on the fact these are the two most common cancer diagnoses for men and women (now revised text in introduction on page 2). Both groups receive treatments rendering a wide range of symptomatic concerns still present during the first year after treatment that need attention through extended support and self-care

management making them suitable for our intervention. We hope that this is now clearer in the background.

---

4. What is Primary treatment? The definition of primary care may vary from country to country. As an article published in an international journal, I suggest you explain this in the article.

Author response:

Primary treatments for breast and prostate cancer are now explained in the manuscript (Introduction page 2). We have also expanded on the text regarding primary care (Introduction, page 3).

---

5. The study is designed as a randomized controlled trial. How to achieve randomization? It was not described in the registered clinical trial, it is recommended to describe it clearly in the article.

Author response:

The studies are cluster randomized into two parallel arms; the intervention plus standard care (intervention group); and standard care alone (control group). The randomization involves entire PCCs that are matched regarding socioeconomic status and size. The process is now described more thoroughly (Trial design, page 5- 6).

---

6. The description in this article differs from the registered clinical trials,

Author response:

We revised the clinical trial registration for the prostate cancer study on September 19th 2024 https://clinicaltrials.gov/study/NCT05100121

(1) The intervention period of this study is 6 months, but the duration of intervention for prostate cancer in the registered clinical trial was 12 months.

The clinical trials registration is updated and now corresponds to the protocol (page 2)

(2) Some of the secondary outcome measures in this paper were not involved in prostate cancer studies in registered clinical trials.

The clinical trials registration is updated and now corresponds to the protocol (page 2)

7.Page 7 line 3
'some studies with a smaller sample size and insufficiently sensitive assessment methods could not detect any changes in QoL. 'it is the limitation of current studies, about your study, have you solve these limitations?

Author response:

This statement from the review of Tuominen et al. 2019 exemplifies a limitation in detecting effectiveness in earlier nursing interventions. It is important to address these limitations in future trials which we hope to do. However, at this stage in our pilot RCTs we don't seek to find evidence of effectiveness (i.e patient QoL) but rather to find out if the design is appropriate to use in a future full-

scale trial. We have now deleted this sentence from the background as it does not really add anything to the rational of our protocol.

Page 16, line 3, 'We have studied the use of Interaktor during treatment for different cancer diagnoses and the result was shown to be very positive...'
In this paragraph, apparently, your study also found no positive effects of the APP in terms of quality of life. Therefore, I suggest that you revise it to make the limitations of the existing studies relevant to your research question and study design.

Author response:

In this paragraph we wrote: *"At two weeks and six weeks after the end of treatment, patients rated better emotional well-being and less symptom distress than the patients in the control groups".* The emotional well-being refers to the HRQoL functional scale in the EORTC-QLQ30. We have elaborated slightly on the text in this paragraph to clarify this (Discussion, page 13)

8.Page7 line 53
'no studies combining ePROs and nursing support in primary care with a focus on improving patient symptom management and QoL'
The expression of this sentence is too arbitrary and it is suggested to be modified.

Author response:

Yes, we agree that this statement and the study rational could be made much clearer. The text is now revised (Introduction, page 5).

9.It is recommended to describe the admission criteria for research nurses.

Author response:

A sentence is now added regarding the nurses who were employed at the PCC and allocated in this project to run the nurse-led intervention (Intervention delivery, page 7).

Reviewer: 2

Comments to the Author:

1. The objective mentions that the study population consists of those "in the first year after receiving primary treatment," but the actual intervention period is 6 months, and the follow-up lasts for 24 months. Isn't this inconsistent?

Author response:

We aim to reach the patients during their first year after finishing treatment. Inclusion criteria for eligibility was having ended primary treatment of breast cancer or prostate cancer within the past six months. We estimated that the intervention period of 6 months would fall within the frame of the first year. This is now clarified, and the text is revised (Participants, page 6).

The follow-up is set for 24-months to see if there are any long-term effects of the intervention and if it is feasible and relevant to have such long follow-up period in the coming RRCT.

2. The inclusion criteria should be more specific. Will patients with comorbidities be included in the study?

Author response:

Yes, patients are asked to state any comorbidities at baseline. Patients can only be included if they have received curative intent treatment that is not having an advanced disease. The inclusion and exclusion criteria have been clarified in the text (Participants Enrolment on page 6)

---

3. Have you considered increasing the sample size?

Author response:

No, we have not considered a larger sample size given the feasibility design of the two trials. The sample size chosen was calculated according to Cocks & Torgerson (2013) sample size calculation for pilot randomized trials using acceptability as a primary measure. This was explained in the sample size paragraph (on page 10) and we have not revised the text further.

---

4. The primary and secondary objectives are somewhat confused and unclear. The primary objective is to assess acceptability and feasibility, but the secondary objectives seem to overlap in scope, particularly regarding the evaluation of potential effectiveness.

Author response:

We agree that this could be made clearer. The aim had now been revised (page 5.) Please also see our response to Reviewer 1 question 2.

---

5. *The methods section lacks detailed descriptions of the randomization and blinding procedures conducted at the primary care centers.*

Author response:

The studies are cluster randomized into two parallel arms; the intervention plus standard care (intervention group); and standard care alone (control group). The randomization involves entire PCCs that are matched regarding socioeconomic status and size. The process is now described more thoroughly (Study design, page 5-6).

It is not possible to blind patients to group affiliation. The blinding only accounts for the researchers through data entry procedure. This is described in the Data management section (page 10) and we have not revised the text further.

---

6. The description of the digital support in the intervention is not detailed enough, particularly regarding the types of recommendations provided through the app and the criteria that trigger alerts.

Author response:

Agree, this should be described much more conclusively. The text is now revised (Nurse-led support, page 8).
